# Analyzing *in situ* gene expression in the mouse brain with image registration, feature extraction and block clustering

**DOI:** 10.1186/1471-2105-8-S10-S5

**Published:** 2007-12-21

**Authors:** Manjunatha Jagalur, Chris Pal, Erik Learned-Miller, R Thomas Zoeller, David Kulp

**Affiliations:** 1 Department of Computer Science, University of Massachusetts Amherst, Amherst, MA-01003, USA; 2 Department of Computer Science, University of Rochester, Rochester, NY-14627, USA; 3 Department of Biology & The Laboratory of Molecular and Cellular Neurobiology, University of Massachusetts Amherst, Amherst, MA-01003, USA

## Abstract

**Background:**

Many important high throughput projects use *in situ *hybridization and may require the analysis of images of spatial cross sections of organisms taken with cellular level resolution. Projects creating gene expression atlases at unprecedented scales for the embryonic fruit fly as well as the embryonic and adult mouse already involve the analysis of hundreds of thousands of high resolution experimental images mapping mRNA expression patterns. Challenges include accurate registration of highly deformed tissues, associating cells with known anatomical regions, and identifying groups of genes whose expression is coordinately regulated with respect to both concentration and spatial location. Solutions to these and other challenges will lead to a richer understanding of the complex system aspects of gene regulation in heterogeneous tissue.

**Results:**

We present an end-to-end approach for processing raw *in situ *expression imagery and performing subsequent analysis. We use a non-linear, information theoretic based image registration technique specifically adapted for mapping expression images to anatomical annotations and a method for extracting expression information within an anatomical region. Our method consists of coarse registration, fine registration, and expression feature extraction steps. From this we obtain a matrix for expression characteristics with rows corresponding to genes and columns corresponding to anatomical sub-structures. We perform matrix block cluster analysis using a novel row-column mixture model and we relate clustered patterns to Gene Ontology (GO) annotations.

**Conclusion:**

Resulting registrations suggest that our method is robust over intensity levels and shape variations in ISH imagery. Functional enrichment studies from both simple analysis and block clustering indicate that gene relationships consistent with biological knowledge of neuronal gene functions can be extracted from large ISH image databases such as the Allen Brain Atlas [1] and the Max-Planck Institute [2] using our method. While we focus here on imagery and experiments of the mouse brain our approach should be applicable to a variety of *in situ *experiments.

## Background

Many large scale molecular biology experiments now use cDNA microarray technology for measuring expression levels of a large number of genes for a small tissue sample or cell. However, there are a number of projects underway to map spatial patterns of gene expression using *in situ *hybridization (ISH) [3] for tens of thousands of genes in different organisms. In contrast to microarray based methods, these projects can produce huge archives of high-resolution 2D and 3D images and involve the analysis of complex spatial patterns of expression in the context of anatomical structures, tissues and cells. These types of ISH experiments are essentially a type of tissue array.

**Figure 1:**
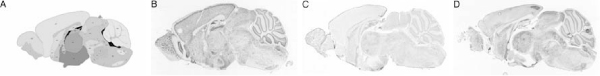
***In situ *hybridization images**. A. Reference image at position 4000. Expression images for B. Abr, C. Adcy5, D. Astn1. B is one of the best quality images. Most of the images are of quality C. D is among the worst quality.

In recent years, genome-wide ISH experiments have started to become publicly available, including: the Berkeley ISH embryonic fruit fly (*Drosophila*) experiments [4], the ISH mouse embryo experiments at the Max-Planck Institute [5], projects at Harvard [6] and Baylor [7] and the extremely large scale ISH experiments of the Allen Brain Atlas [1,8], involving over 21, 000 genes and roughly three hundred 5000 × 5000 pixel images per gene for the adult mouse brain. The processing and analysis of ISH experiments, the linking of atlas based experimental archives with relevant scientific literature, and the comparison of results with existing knowledge, together have the potential for tremendous impact on the scientific community. In our experiments here we focus on the processing and analysis of ISH experiments of the adult mouse brain using data from the Allen Brain Atlas [1,8] with properties very similar to the the Max-Planck data [5]. Figure 1 shows some examples of the imagery from the Allen Brain Atlas.

We concentrate here on extracting expression information from the type of imagery typically found in these resources. To achieve this we have developed algorithms to register each gene expression cross section to a corresponding reference image. We then use these warped images to estimate the expression characteristics of a gene across different anatomical structures and extract biologically meaningful information from this data through a simple enrichment analysis, as well as with a block clustering algorithm.

In both the Allen Atlas and the Max-Planck data, images are of very high resolution (roughly 5000 pixels per inch) while the number of slices through the brain for a given gene expression experiment is moderate for the Allen Atlas (≈300) and small (≈10) for the Max-Planck data. The intensity of each pixel gives an estimate of the expression of that particular gene at that location. Basic searching tools have been integrated into the BrainAtlas that help users search for genes expressed in specific parts of the brain [1].

Since our goal is to gather statistics about common expression patterns in anatomical structures across experiments, it is important that we achieve a registration that is as accurate and robust as possible. In most of our experiments, we align each expression image to a hand drawn anatomical reference image. This hand drawn reference was created by an anatomist referring to their Allen Reference Atlas, which consists of Nissl stained sections of an unfixed, unfrozen mouse brain [9]. The reference image shows major anatomical structures as fixed colored regions. Several properties characterize the specific nature of our registration task and influenced our approach to the registration problem:

• Our images have very high resolution. Because of this, we are frequently able to find a large number of points in the registration for which we have high confidence. In other words, there is a lot of data for establishing correspondences in certain areas. This suggests using a method that uses a combination of local registrations. Such an approach may not be possible in registration problems using images of lower resolution, since there may not be enough information in the images to have high confidence for a large number of registration points.

• Our images have undergone large non-linear distortions. Because of this, and the size of the images, the space of possible transformations is very large relative to some other registration problems, such as intra-patient registrations in medical imaging. Searching this transformation space directly, either by trying all possible transformations, or through iterative optimization methods, is not feasible: there are simply too many transformations, and a program designed to do this would take prohibitively long to run. Iterative optimization methods like gradient descent, in which a small change is made to the current transformation at each step to improve the results, is likely to get caught in local optima, where all small changes make the result worse, and yet a good transformation has not yet been found. These difficulties further point toward the use of a piecewise, or local, registration process.

• Finally, we wish to have a fully automatic registration procedure. While the first two points above suggest landmark based registration methods, we would like these landmarks to be selected automatically, rather than manually as occurs in many methods.

Together these considerations led us to the development of a fully automated, piecewise, landmark-based registration method. Below we discuss our method in the context of other registration work, and we give details of our approach in the section on methods.

Analysis of the resulting data suggests that this approach can yield biologically meaningful information. Many genes had high expression values in the organs consistent with their known function. Furthermore, our novel, probabilistically principled block clustering algorithm also discovers biologically meaningful clusters. To summarize, the contributions of this paper are: (a) a novel information theory-based landmark algorithm to register images; (b) extraction of expression values; and (c) analysis of these expression values to generate biologically-motivated hypotheses.

### Previous work

#### Registration and feature extraction

Registration of medical and biological images is a heavily studied topic with dozens of distinct approaches and a huge literature. General surveys of registration include those by Toga [10] and by Maintz and Viergever [11].

Our registration procedure relies on automatically identified landmarks, and then bases a global registration on a piecewise landmark-based registration. This means that certain landmarks which are deemed to be in correspondence in the two images are "pinned" to each other and the remaining parts of the images are stretched to fit amongst these pinned landmarks. The most closely related method of which we are aware, developed for multi-modal image analysis, is from Gopalakrishnan et al. [12], which finds information rich landmarks automatically and uses an approximate local affine (The term *affine registration *refers to a registration in which one image has undergone an *affine transformation*. An affine transformation is an image transformation in which straight lines remain straight, and parallel lines remain parallel. In other words, it refers to a registration without the introduction of curvature or perspective distortion. Affine registrations include shifts (or "translations"), rotations, scaling (magnification and reduction along each axis), and *shearing*. While it is not technically correct, affine transformations are sometimes referred to as linear transformations. In this work, we consider a subset of affine transformations that include all of the above operations except shearing.) registration using these landmarks. Although there are many differences in the details, the methods are similar in spirit. Another very similar strategy has been used by Pitiot et al. [13] to analyze histological sections.

Our criterion for alignment is based upon mutual information, as in the original work by Maes et al. [14] and by Viola and Wells [15]. Mutual information is a common criterion of alignment, and has been used heavily in registration algorithms. Intuitively, mutual information alignment is similar to correlation based methods, in which one image is warped until the brightness values at each location correlate as strongly as possible with the brightness values in the other image. Mutual information registration works on a similar principle, but rather than striving to maximize *linear dependence *among pixel values, as in correlation methods, mutual information methods strive to maximize general statistical dependence, both linear and non-linear. Mutual information is, in fact, a numerical measure of this statistical dependendence between the pixel values in two images. We discuss how it is computed for a pair of images in the methods section. The idea, then, behind mutual information registration is that, when images are registered (or aligned) as well as possible, the pixel values in the images will have the strongest possible statistical dependencies. An extensive survey of mutual information alignment algorithms has been published by Pluim et al. [16].

Other work related to our goals here has sought to construct 3D models from high resolution optical photography of post-mortem human brain slices [17] or registration of mouse brain histology from a Nissl stain to obtain a reference volume [18]. In contrast, here we are interested in obtaining registrations between a reference volume and gene expression experiments, potentially a more challenging problem. Recently, Ng et al. [9] explored a strategy for ISH to Nissl registration in which a high resolution B-spline grid is used to obtain a deformation field that warps a Nissl reference onto a subject image. For their local matching cost for the deformation they used a weighted combination of a mean squared difference of a tissue mask and the mutual information of image intensities. They note that the problem is challenging because the expression data is by nature, regionally variable and of varying intensity. Further, they suggest that landmark based approaches show future promise for this setting. These observations further motivate our approach here.

Recent attention has also been given to the processing of less complicated, high resolution *in situ *images of drosophila embryos [19]. In such cases, the registration step is fairly straightforward because the embryonic shape is simple and smooth; basic affine transformations appear to lead to registrations of satisfactory accuracy for early stages of development.

#### Spatial expression clusterin

In this work, we frame the analysis of extracted expression levels in terms of a clustering problem in two dimensions: genes and anatomical structures. Approaches to such clustering include independently grouping the rows and columns of the data matrix [20] (ignoring any dependencies between the two clustering problems) and *bi-clustering *[21], in which both the rows and columns of the matrix are simultaneously clustered. This setting can lead to a coupling of the two clustering procedures [22]. These methods have been widely applied to microarray data [23] as well as other heterogeneous data [24]. Earlier related work on *direct clustering *[25] considered finding joint row and column clusters or blocks. More recently, [26] have cast the joint row and column clustering problem as a *block mixture model*. Here we present a novel block mixture model and novel algorithms for optimizing the model. Using these methods we perform block cluster analysis of expression levels extracted for anatomical structures.

## Results and discussion

To test the viability of our system we collected several mid-sagittal section images from the Brain Atlas for each of 104 genes and also collected five reference brain images from the same region. The resolution of each image (Figure 1) is 400 × 800 pixels. While the raw imagery in the atlas has a purple color, we work with images in "gray scale", which means each pixel is defined by a single numerical value representing its brightness, and there is no color information. Registration between a reference image and an expression image was performed in two stages: coarse registration and fine registration.

### Registration

**Figure 2:**
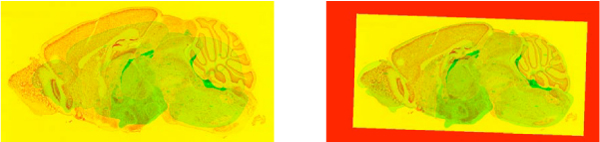
**Approximate registration**. Images show the difference between expression image and reference image before(left) and after approximate registration. In these images red channel corresponds to reference image and green channel corresponds to expression image.

The coarse registration step is done to put the ISH image in rough alignment with the reference image. Figure 2 shows the result of the coarse registration step. The main purpose of this step is to ease the computational burden on the fine registration step (discussed below). In particular, if the images are in rough alignment, the fine registration step can assume that a pair of corresponding points in the reference image and the histological image are at similar locations. To obtain a rough alignment, a *global *affine transformation (one affine transformation for the entire expression image) is done between the reference image and the expression image. More details are given on this step in the methods section.

Once a coarse registration has been done, a more accurate fine registration is performed. This fine registration consists of five steps.

**Figure 3:**
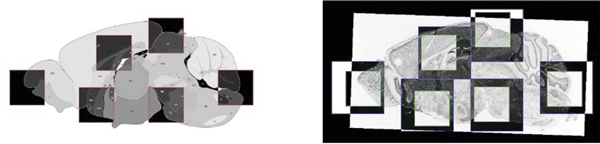
**High entropy landmarks**. Squares on the left image highlight some of the high entropy landmarks in the reference image. Large squares on the right image show the search space for those landmarks in the expression image, and small squares show the optimal patch that corresponds to those landmarks.

**Figure 4:**
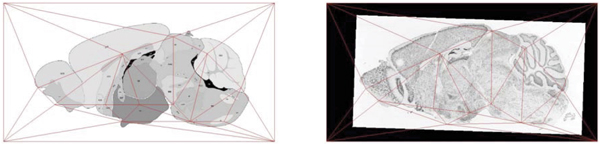
**Delaunay triangulation of landmarks**. The left image shows the Delaunay triangulation based upon a set of landmarks in the reference image. The right image shows the corresponding triangulation of mapped points in an expression image. Triangulation in the right image might not be the Delaunay triangulation.

1. In the first step, which is only performed once per reference image, points in the reference image that are "distinctive" are selected as a basis for the alignment. The goal is to find a set of points which can be matched with corresponding points in the histological image with high reliability. The measure of distinctiveness is the entropy of the neighborhood of the point. The entropy can be thought of as a measure of the complexity of a point's neighborhood in the reference image. Neighborhoods with high entropy (such as the junction of three different anatomical structures) are likely to have more structure to provide a local and repeatable match. Neighborhoods with low entropy (such as a neighborhood around a patch of constant brightness) do not have enough structure to provide an unambiguous match. The left side of Figure 3 shows some of the high entropy neighborhoods selected in the reference image as distinctive landmarks for registration.

2. Once a set of distinctive points have been found in the reference image, the next goal is to find the corresponding points in the expression image. This is done by searching a small neighborhood in the expression image for the best match to the reference image. Because the two images are roughly aligned from the previous step, the search for the best match can take place over a smaller zone in the expression image. The right side of Figure 3 shows the matched landmarks in the expression image.

3. Using the landmarks in the reference image (from the first step), the next step is to define a set of triangular regions in the reference image that will be individually registered to corresponding regions in the expression image. To do this, a "triangulation" of the reference image, using the set of identified landmarks, must be performed. We do this using a standard procedure known as Delaunay triangulation, which is described further in the methods section. Intuitively, a Delaunay triangulation is designed to break the image into triangles such that "sliver-like" triangles are avoided as much as possible. The left side of Figure 4 shows a Delaunay triangulation of the reference image based upon the landmarks which have been defined at each triangle vertex.

4. Once the reference image has been triangulated, the corresponding triangulation of the expression image is formed. It may be necessary to eliminate some reference points in order to keep the new triangulation from containing crossed lines (and thus ambiguous regions). Details on this culling procedure are given in the methods section. The right side of Figure 4 shows a typical triangulation of an expression image.

5. At this stage, the algorithm has established correspondences among triangles in the reference image and the expression image. The pixels within each triangle of the expression image are then warped according to a bi-cubic interpolation scheme (explained in methods section) to match the pixels in the reference image.

**Figure 5:**
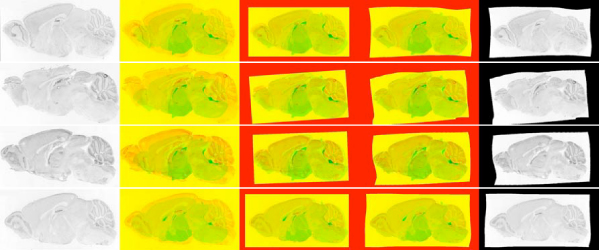
**Well registered images**. First column corresponds to the raw image. Images in the subsequent columns show the difference between reference image and raw image, image after approximate registration and fine registration respectively. In these images red channel corresponds to reference image and green channel corresponds to expression image. Note that as in these images (reference and expression) high intensity (or white) corresponds to low/no expression and vice versa. The last column has the registered image. Rows correspond to genes *Cpeb1 *(Maximum mutual information = 0.5962), *Esr2*(0.6118), *Rasd2*(0.6285) and *Tdo2*(0.6209) respectively. About 55% of the registrations were of this quality.

**Figure 6:**
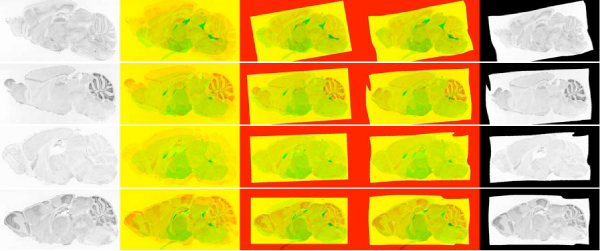
**Moderately well registered images**. Rows correspond to genes *Bbs4*(0.5721), *Chd7*(0.5325), *Pde1b*(0.4894) and *Sphk2*(0.5909) respectively. About 40% of the registrations were of this quality.

**Figure 7:**
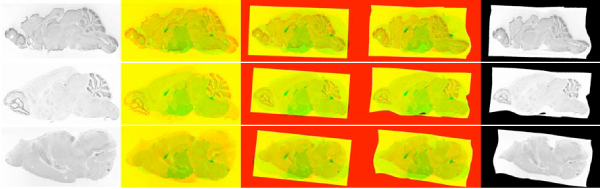
**Poorly registered images**. Rows correspond to genes *Apaf1*(0.5387), *Reln*(0.4720) and *Myo5b*(0.5148) respectively. About 5% of the registrations were of this quality. First two of these images have large deformation (their medulla is elongated) that couldn't be reconciled with our approach. The third image is rotated and flipped. Our algorithm does not consider this type of transformation for computational reasons (i.e it would blow-up the running time).

**Figure 8:**
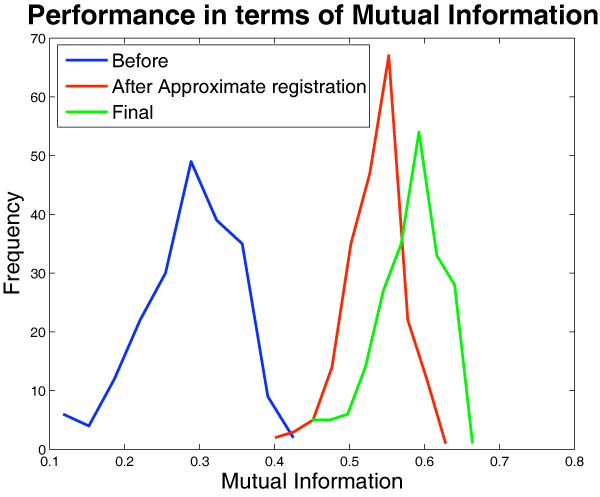
**Quantitative performance of registration**. Density plot of mutual information between test and reference images before approximate registration, after approximate registration, and final registration.

**Figure 9:**
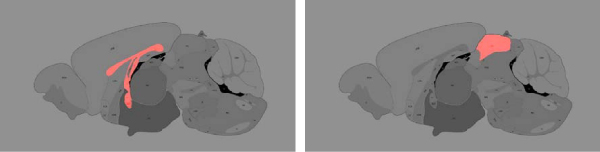
**Examples of anatomical structure masks**. Anatomical masks (highlighted in pinkish-orange) for *hippocampal area *and *superior colliculus*.

All images for each gene were registered against all five reference images and the best pair was selected according to maximum mutual information. Figures 5, 6 and 7 show the resulting images at various steps of registration. Analytical results of the registration is presented in Figure 8. Masks (examples shown in Figure 9) were created for each anatomical structure that was labeled in the reference image, allowing for the corresponding pixels to be extracted for each feature (examples shown in 10). Further analysis was done on these extracted features to provide biological validation of the methods (Figures 11 and 12). Detailed results are provided below.

**Figure 10:**
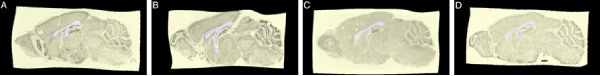
**Examples of anatomical structure expression extraction.  **Gene expression images with hippocampus mask overlaid in blue.  Corresponding genes are A. *Abr*, B. *Aplp2*, C. *Aff2*, D. *App*. For a given pixel intensity value I, expression values are calculated via  (256-I)/256. The 75 percentile of expression values of all pixels in a  mask are then used as the expression statistic.

**Figure 11:**
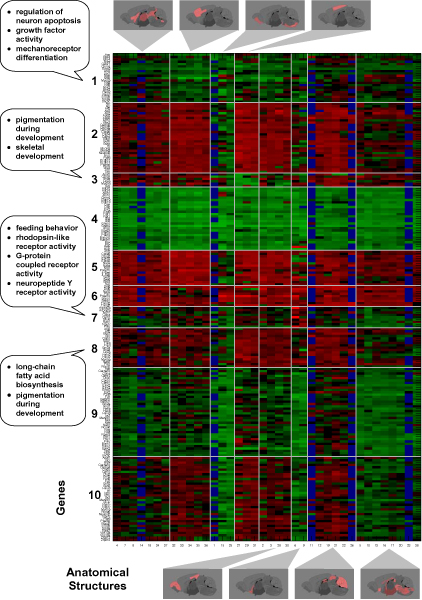
**Data after block clustering and analysis of the formed clusters**. Expression data block clustered using 10 × 8 row-column mixtures model. Each of the block is defined by a set of genes and set of anatomical structures. Missing values, indicated in blue, result from the absence of certain  anatomical structures in the corresponding images. Red corresponds to  high expression and green corresponds to low expression.

**Figure 12:**
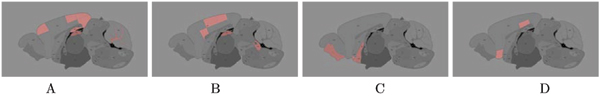
**Enrichment for GO terms**. Regions enriched (p-value < 0.01) for expression of genes involved A. sensory perception of sound (GO:0007605), B. learning and memory (GO:0007611), C. feeding behavior (GO:0007631), and D. visual learning (GO:0008542).

**Figure 13:**
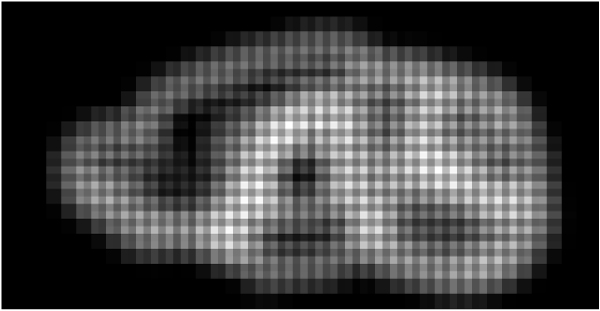
**Distribution of high entropy patches**. In this image intensity at each point is proportional to average entropy of 50 × 50 blocks overlapping that point.

Figure 5 shows some well registered images with almost all the anatomical structures aligning correctly. Approximately 55 out of 104 images are of this quality. Figure 6 shows moderately well registered images with a few misaligned anatomical structures. Around 40 out of 104 images are of this quality and are sufficient for coarse feature extraction. From these results it appears that image registration is successful under varying shape, intensity levels and quality. Figure 7 shows some cases where the registration algorithm failed. 5 out of 104 images were of this quality. On manual examination, it is evident that these images cannot be registered using a continuity preserving local transformation. Figure 8 plots the density of mutual information between test and reference images at various points of registration, which further shows that the image registrations for a large majority of genes is successful.

### Feature extraction: estimation of expression values

Individual "anatomical structure masks" were constructed for each of the brain regions annotated in the reference images (cerebellum, cortex, etc.). Figure 9 shows some of these masks. Each of these masks were applied on the corresponding registered image for each gene to extract pixels for that structure. Figure 10 shows some of the registered expression images masked for specific structures. While most images are not sufficiently well-registered to do a pixel-level feature extraction, gross features like mean, median and quantile for expression levels across the entire structure can be extracted with a high reliability. In the following analysis we chose to use the 0.75 quantile value of these pixel intensities as the expression statistic, which we found to be more robust to outlier effects due to registration errors and heterogeneity within the structure.

Lein, et al. [8] reported that expression levels were typically consistent within structures in the mouse brain. For example, they note that expression levels are "relatively uniform ... across all cortical areas, consistent with the idea that the basic (canonical) microcircuit is conserved across the entire neocortex." It is these sorts of structural effects that we aim to capture in our feature extraction. Nevertheless, Lein also emphasize that there are notable examples of expression sub-structure, such as varying expression among hippocampal sub-regions. Figure 13 shows this phenomenon in the hippocampus. Automated identification of such sub-regions and the correlation of genes within these sub-regions is an area for future improvement.

**Figure 14:**
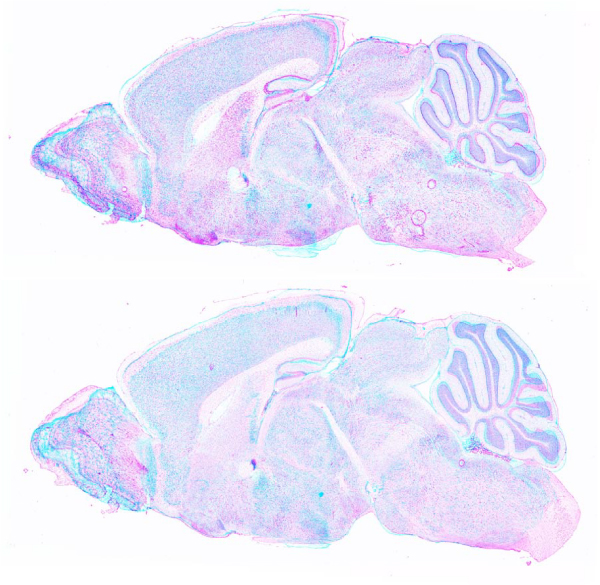
**High resolution image registration**. First two images show the registration between high resolution images for genes *Chst2* and *Sfrp2* (green channel) with that of *Nr2f1* (red channel). Blue channel is set to maximum blue intensity.

In this paper, we limit our concern to the major annotated structures, which allows for a consistent set of biologically meaningful structures across all gene expression experiments. This naturally yields a matrix of anatomy-by-gene expression level. Figure 11 shows a heat map where columns correspond to the anatomical structures and rows correspond to genes. Red indicates high expression and green, low expression.

### Scaling to high resolution

A key question is whether the registration and feature extraction techniques are scalable to higher resolution images. Although not publicly available at the time of these experiments, the Allan Brain Atlas images, for example, have original dimensions of ≈5000 × 10000 pixels. To begin to address this question, we obtained a small amount of data directly from the Allen Institute as well as additional high resolution mouse brain imagery from the Max-Planck Institute's GenePaint database. In this case, we do not have hand-annotated reference maps of anatomical structures and so instead we treat an arbitrary image as a reference and consider the pairwise registration quality between ISH images.

**Figure 15:**
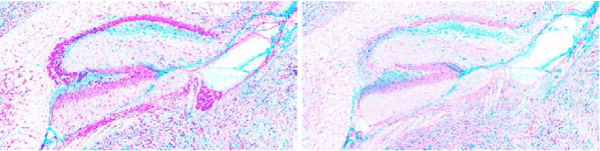
**Closer view of hippocampal region**. Hippocampus plays a very important role in memory and spatial navigation. This figure shows the registration in this region.

**Figure 16:**
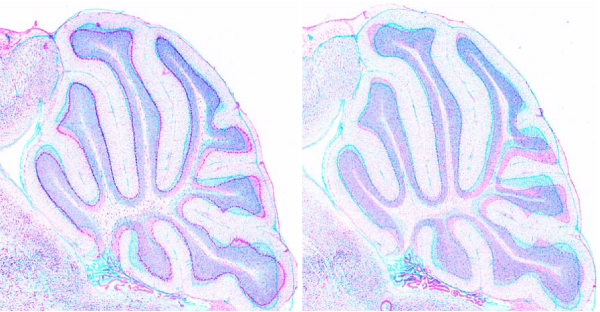
**Closer view of cerebellum region**. Cerebellum plays an important role in sensory perception and motor output. This figure shows the registration in this region.

Sample results from these experiments are presented in figures 14, 15 and 16. Table 1 contains the performance of our algorithm in terms of mutual information. Figure 15 shows that delicate anatomical structures, such as the hippocampus, can be successfully assigned.

**Table 1:** Mutual information between high resolution images.

Gene1	Gene2	MI Before	MI after coarse reg.	MI after fine reg.
Sca1	Nr2f1	0.030602	0.192711	0.211132
Chst2	Nr2f1	0.053200	0.300494	0.334058

### Functional analysis and block clustering

There is substantial enrichment of brain-related gene function in specific anatomical structures. For example, in figure 12.B, genes associated with learning and memory (GO:0007611) were found to be highly expressed in the upper parts of the cortex and the medial habenula, just below the hippocampal formation (p-value 0.001). In another case, feeding behavior genes (GO:0007631) were found to be highly expressed in the lower part of the olfactory bulb (figure 12.C, p-value 0.008). In the same figure, additional examples of spatial enrichment can be seen for brain-related gene functions related to sensory perception and visual learning.

Using a novel block clustering technique described in the methods section, below, we permuted the rows (genes) and columns (anatomy) to group correlated regions and gene sets. We believe that this clustering assists us in identifying biologically meaningful information about genes and anatomical structures. We justify this by noting that many genes within block clusters have high expression values in organs consistent with their known functional annotations. For example, when a coarse scale 5 × 5 block clustering is applied to our data, we find a high expression block for the cerebellum and the cortex with gene clusters containing Aff2, Prkar1b, Shc3, Tmod2, Abi2, all of which are associated with learning and memory (p-value 0.001). Figure 11 displays the block clustering results of a 10 × 8 class model, which demonstrates that *block constant patterns are indeed present within the data*. We use a false color image with scaling in a format commonly used in microarray visualization, high expression levels are red and low expression levels are green. Missing values arising from different anatomy and slice cross sections are indicated in blue.

Both the variational and sequential optimization methods described in the methods section for block clustering with a row-column mixture model produce comparable and improved quality clustering results in comparison to independently applied row and column clustering methods and a variety of other optimization algorithms [27]. Here we used the sequential method for the results shown in figure 11 as our implementation runs faster for matrices of the size considered. We used the best result over 10 runs for a 10 row and 8 column class model. Table 2 shows the clusters from figure 11 that are enriched for genes associated with a particular GO category with p-values ≤ 0.001. We contrast this with table 3 in which we used the same enrichment analysis for independently applied clustering on rows and columns for a traditional mixture model. From this comparison we see that the joint clustering of the row column mixture has allowed us to obtain more clusters enriched for biologically meaningful GO categories.

**Table 2:** Clusters discovered with a joint row column mixture model (block clustering). Type corresponds to either (P)rocess or (F)unction.

Clust	GO number	Type	p-value	Description	Genes
1	0008083	F	.0091	growth factor activity	Bdnf, Ntf3
1	0042490	P	.0037	mechanoreceptor differentiation	Bdnf, Ntf3, Ntrk2
1	0043523	P	.0091	regulation of neuron apoptosis	Bdnf, Ntf3
3	0001501	P	.0055	skeletal development	Gnaq, Hexa
3	0048066	P	.0004	pigmentation during development	Gnaq, Myo5a
7	0001584	F	.0010	rhodopsin-like receptor activity	Adora2a, Mc4r, Npy1r, Prlhr
7	0004930	F	.0037	G-protein coupled receptor activity	Adora2a, Mc4r, Npy1r, Prlhr
7	0004983	F	.0055	neuropeptide Y receptor activity	Npy1r, Prlhr
7	0007631	P	.0010	feeding behavior	Calca, Mc4r, Npy1r, Prlhr
8	0042759	P	.0091	long-chain fatty acid biosynthesis	Myo5a, Plp1
8	0048066	P	.0091	pigmentation during development	Gnaq, Myo5a

**Table 3:** Clusters discovered with a traditional mixture model (independent clustering). Type corresponds to either (P)rocess or (F)unction.

Clust	GO number	Type	p-value	Description	Genes
A	0007166	P	.0002	cell surf. receptor linked sig. trans.	Erbb2, Fzd9
B	0007631	P	.0062	feeding behavior	Calca, Mc4r, Npy1r, Ntrk2
C	0001501	P	.0092	skeletal development	Gnaq, Hexa
C	0048066	P	.0007	pigmentation during development	Gnaq, Myo5a

For practical reasons due to data availability and ease of analysis, we focused on a relatively small set of 104 well-annotated genes and limited our consideration of structures to the major anatomical features defined in the reference maps. Since additional spatial sub-structure clearly exists, future work requires scaling up the clustering to include hundreds or thousands of sub-structures and incorporating all (≈20, 000) gene expression hybridizations. Based on previous work [27], we expect that hard assignment, variational methods will have superior running times to sequential methods in these larger matrices. Thus, the methods we have developed here should be applicable to other ISH image collections and should scale to larger image sets of higher resolution.

Room for improvement certainly remains. Although we have shown reasonable block constant structure is present in the data matrix, recently developed, related methods allowing overlapping groups such as Matrix Tile Analysis [28] and other bi-clustering methods [23,29] may yield future insights. A potentially more important extension of this approach is to use more sophisticated feature extraction methods to obtain richer cellular level information. Such methods could capture expression properties related to cell type such as neurons, astrocytes, oligodendrocytes and others. For example, our subset of experimental images exhibited striking expression differences in the Purkinje cell layer of the cerebellum.

## Conclusion

The high mutual information gain in our image registration scheme (figure 8) along with manual review suggests that the registration method is largely successful. From figures 5 and 6 it is evident that anatomical structures can be registered reasonably well even when there is a large variation in shape or there is deletion of parts or presence of debris. Figures 10 and 15 show that assignment of very delicate anatomical structures, such as the hippocampus, are often successful. However the variation in image and sample quality is high, leading to difficult cases that probably cannot be adequately registered under any continuity preserving transformation.

We found functional enrichment among anatomical structures, as expected. But more generally, we demonstrated how our novel block clustering strategy can extract block constant structure and is likely to also scale well to larger problems.

## Methods

As described in the results section, a gene expression image is first coarsely registered to a corresponding reference image using a single affine transformation. Later fine registration is done by matching the high entropy landmarks between the two images. Then using the registered version of the gene expression image, gene expression values are extracted and analyzed. We now present details of each step.

### Coarse registration

In our approach we start with a Nissl stain reference image with hand annotated anatomical regions (Figure 1A). For each experimental ISH expression image, we perform a coarse registration using a global affine transformation (Figures 5, 6 and 7, middle column). Parameters of this affine transformation are optimized sequentially. We start with a set of initial parameters and each parameter is optimized in isolation by searching over a set of values to (Initially rotation is set to zero, offsets are set to zero and scaling is set to one. Then horizontal scaling is optimized by shrinking or expanding by 0 to 70 pixels, followed by vertical scaling (shrinking or expanding by 0 to 50 pixels), followed by rotation by 0 to 30 degrees on each side, followed by horizontal offset, and finally vertical offset. (offsets are 0 to 50 pixels in both directions.) This step was repeated until none of the parameters changed. Usually convergence was reached within five iterations. Note that these transformations are applied on a 1/5th scaled down images.) maximize *mutual information *(discussed in detail below) between the transformed expression image and the reference image for each transformation. We then select the transformation resulting in the greatest mutual information.

The *absolute quality *of the registration cannot be assessed, in general, by examining the mutual information score. This score only gives a relative measure of how different transformations compare. But coarse registration is relatively easy as the image does not contain other objects and the global shape of the two-dimensional sections is usually similar in the two images. The main objective of this step is to reduce the size of the set of possible transformations (i.e., the *search space*) for the fine registration. After coarse registration, we expect the anatomical structures to be in similar locations, and to have similar size and orientation, in the two images. To make this step computationally viable, images are scaled down by a factor of five in each dimension.

### Fine registration

As the exact shape of the brain varies between the images, a global transformation cannot capture the morphing needed to align expression and reference images. But as we have already peformed a coarse registration, individual anatomical structures are mostly aligned and require only local transformations for better alignment. We achieve this by using a five-step, information theory-based landmark extraction and matching approach. In the first step (1), high entropy patches, which are likely to contain more information about shape and structure, are extracted. Then (2) the coarsely registered expression image is searched locally for patches that match each of the distinctive landmarks in the reference image. After the triangulation (3 and 4) of each image (explained below), these mappings are used to perform a bi-cubic interpolation (5) between the two images to obtain an accurate alignment. Using a 2 GHz processor the complete coarse and fine registration procedure takes approximately 20 minutes to register 400 × 800 pixel imagery and about 1 hour to register the higher resolution, 5000 × 5000 pixel imagery.

#### Landmark extraction

The goal of landmark extraction is to find patches in the reference image which can be matched in the expression image with relatively high confidence. The intuition behind our method for selecting distinctive patches for matching is as follows: the more "structure" that one sees in a patch, the lower the chance that it will be spuriously matched to a patch in the expression image. For example, if a patch in the reference image is completely constant (in brightness), then we cannot be confident we have matched it correctly to a patch in an expression image. Hence, we want a notion of "complexity of structure" in the reference image for patch selection. We use the *entropy of the distribution of brightness values in a patch *as a measure of this complexity. The formal definition and calculation of entropy are discussed below.

75 high entropy 100 × 100 pixel patches are identified in the reference image, which are then used to define anchor locations within each expression image for our refined registration. Patches may overlap, but we limit the maximal overlap to be 50%. That is, we select patches in order of entropy, skipping patches which overlap previously selected patches by more than 50%.

High entropy regions often correspond to edges or places with high anatomical variation. This is reflected in Figure 13 which contains a two-dimensional histogram of high entropy square patches around the image. A similar concept called saliency [30] has been proposed and used to extract information-rich features from images.

#### Landmark matching

The anchor patches are more precisely mapped to the experimental image by performing a local search over a small subset of affine transformations in a 150 × 150 pixel window defined by the initial anchor position in the reference. For a fixed set of affine transforms, we warp a small patch in the expression image using that transform, and compare it to the anchor patch in the reference image. The set of transformations used is represented by all combinations of the following simpler transformations: shrinking or expanding by one or two pixels in each direction; rotation by one or two degrees in each direction; shifting anywhere from 0 to 25 pixels (in steps of 5 pixels) in each direction (up, down, left and right). Notice again that these transformations do not include the possibility of shearing. However, here again we use a local mutual information based matching criterion. After local registration, the centers of the patches in the reference and experimental images serve as the key pixel correspondences between the images.

Every patch selected in the previous step is then mapped to its corresponding patch in the gene expression image. The selection and mapping of these landmarks is illustrated in Figure 3. The center of each of these patches are used as landmarks to register between images. As some parts of the image look very different from the reference images, it is likely that all of the matches are correct. Global consistency using Delaunay triangulations, described next, are used to filter such patches.

#### Delaunay triangulation

A triangulation is a decomposition of an image into triangles based upon a set of points that form the vertices of the triangles, as shown in Figure 4. Triangulations are often used to break an image into simpler parts, especially when the reference points do not occur in a regular grid (like a rectangular grid). A *Delaunay triangulation *is a particular type of triangulation that minimizes the number and size of small angles in the resulting triangulation. See [31,32] for details and algorithms to construct Delaunay triangulation. The scientific package Matlab has library routines for creating Delaunay triangulations as well.

Using the landmark points established in the first step, a Delaunay triangulation of the reference image is constructed. The goal of the fine registration is to register each triangle of the expression image to one of the triangles in the reference image (see Figure 4).

#### Matching the triangulation to the expression image

The Delaunay triangulation of the reference image, made from the landmark points, defines a set of triangles in the image. In particular, it says which triples of landmark points should be connected to form regions, as in Figure 4. If, for example, the landmark points in the reference image are labeled {*A*, *B*, *C*, *D*, *E*,...}, then the Delaunay triangulation could be expressed as a set of triples of these landmarks, such as {[*A*, *B*, *D*], [*B*, *C*, *D*], [*A*, *D*, *E*],...}.

We would like to form a triangulation in the expression image using the corresponding triples of landmarks that we had used in the reference image. For example, if the landmark points in the expression image are labeled {*A'*, *B'*, *C'*, *D'*, *E'*,...}, then we would like to form the following triangulation: {[*A'*, *B'*, *D'*], [*B'*, *C'*, *D'*], [*A'*, *D'*, *E'*],...}. Unfortunately, this may not always be possible, since the points in the expression image are in slightly different positions than their correspondents in the reference image. In particular, edges of the triangles in the expression image may cross and create ambiguous regions. To eliminate these regions, we simply remove a landmark if it appears within another Delaunay triangle in the expression image. This process is repeated until none of the triangles overlap. Every time a landmark is deleted Delaunay triangulation is recomputed with the remaining landmarks.

In an additional step, all the landmarks that are responsible for straining the mapping are removed. Specifically, if *AB *is an edge in the triangulation of the reference image and *A'B' *is the corresponding edge in the expression image, and if the ratio of the lengths of these edges is either greater than 1.5 or less than 0.67, then one of the points is removed. This procedure alleviates some additional bad matches in the correspondence process and improves overall registrations. In this step also every time a landmark is deleted Delaunay triangulation is recomputed with the remaining landmarks.

#### Bi-cubic interpolation

At this point, we have established a triangulation in the reference image and a corresponding triangulation in the expression image. The only remaining step is to map each pixel in the expression image to a new location, using the corresponding landmarks of the surrounding triangle as reference points. This is done using an interpolation scheme known as bi-cubic interpolation [33]. The bi-cubic interpolation is used instead of linear interpolation to preserve smoothness of the transformation.

### Calculating entropy and mutual information

We use discrete entropy as a measure of the complexity of an image or image patch. It is a function of the brightness values of a single image. We use mutual information as a measure of the relative goodness of matching between two images or two image patches. It is hence a function of the brightness values of two images.

Let *X *= {*x*_*ij*_} be the set of intensity values of the pixels in the image *X *and *Y *= {*y*_*ij*_} be the intensity values of the pixels in image *Y*. The value of pixel intensities are in the range [0, 255] which can be divided into 16 equal intervals {*V*_1_, *V*_2_,⋯,*V*_16_}. Histograms of images X and Y, and their joint histogram, can be calculated by counting the number of pixels falling in each interval.

$$\begin{array}{c} C_{k}^{X}=Count(X_{ij}\in V_{k})\\ C_{k}^{Y}=Count(Y_{ij}\in V_{k})\\ C_{kl}^{XY}=Count((X_{ij}\in V_{k}))AND(Y_{ij}\in V_{l})) \end{array}$$

Here *Count *is a function that counts the number of instances that the argument condition is satisfied. Using these histograms, the probability and joint probability distribution of the images can be calculated:

$$\begin{array}{c} P_{X}(V_{k})=\frac{C_{k}^{X}}{\sum_{m}C_{m}^{X}},\\ P_{Y}(V_{l})=\frac{C_{l}^{Y}}{\sum_{n}C_{n}^{Y}},\\ P_{XY}(V_{k},V_{l})=\frac{C_{kl}^{XY}}{\sum_{m,n}C_{mn}^{XY}}. \end{array}$$

The entropy of *X *is calculated using

$$H(X)=-\sum_{k}P_{X}(V_{k})\log P_{X}(V_{k}).$$

Mutual information between images X and Y is calculated using

$$I(X;Y)=\sum_{k,l}P_{XY}(V_{k},V_{l})\log\frac{P_{XY}(V_{k},V_{l})}{P_{X}(V_{k})P_{Y}(V_{l})}.$$

One interpretation of the mutual information between two random variables X and Y (here defined by the brightness values in two different images) is as the *relative entropy *between the joint distribution and product distribution. Or it is equal to difference between the sum of individual entropies and the joint entropy:

$$\begin{array}{lll} I(X;Y) & = & D(p(x,y)||p(x)p(y))\\ & = & H(X)+H(Y)-H(X,Y)\\ & = & \sum_{x,y}p(x,y)\log\frac{p(x,y)}{p(x)p(y)}. \end{array}$$

The last equation is interesting because the fraction in the summation becomes unity, the log of which is 0, and the mutual information becomes 0, when *X *and *Y *are independent random variables. In other words, for independent random variables, the mutual information is zero. Conversely, when two sets of values (like the brightness values in two images) are highly dependent, the mutual information will be relatively large. This is the justification for using it as a measure of the quality of alignment in registration.

### Analysis of expression levels

Once the expression images are registered, the annotated anatomical regions in the reference image are mapped to the experimental image, allowing for the expression levels within each region to be easily extracted in an automated manner. To achieve this we use colored "masks" to delineate anatomical boundaries in our reference imagery, we can compute a variety of feature measurements for each anatomical structure in a straightforward and semi-automated manner. These procedures are then used to extract pixels associated with the corresponding anatomies from every registered expression image. We have experimented with simple summary metrics for each anatomical region such as the mean and median expression level, however, we have found that a robust, quantile measure results in superior performance. Figure 11 shows the 75th percentile for each of 38 anatomical regions for 104 genes. These values have been obtained and summarized in a matrix of genes and anatomies that can be analyzed in a very similar way as analyzing gene expression data obtained in a microarray experiment.

For functional enrichment analysis of individual anatomical regions, the five most highly expressed structures were identified for each gene and it is assumed that the gene is over-expressed in those structures (lack of normalization data prevents us from using a global threshold).

### Functional enrichment

For single gene/structure enrichment as well as enrichment in block clusters, we use the Gene Ontology [34] terms associated with each gene, producing frequencies relating the number of occurrences of each GO term to each structure. We then tested for statistical over-representation of GO terms in each structure (p-value < 0.01) using the hypergeometric distribution (Fisher exact test).

Specifically, the hypergeometric distribution provides the probability of observing exactly *n *genes within a group of *K *genes by chance being associated with a GO category containing *C *genes from a total of *G *genes being analyzed. For our experiments p-values are given by $p=1-\sum_{i=0}^{n}\left(\begin{array}{c} C\\ i \end{array}\right)\left(\begin{array}{c} G-C\\ K-i \end{array}\right)/\left(\begin{array}{c} G\\ K \end{array}\right)$. This test indicates whether a cluster is enriched with genes from a GO category to a greater extent than would be expected by chance.

### Block cluster analysis

Clustering is a widely used tool in bioinformatics. We present and apply a novel, probabilistically principled approach to block clustering in which row and column clusters influence one another. The problem is formulated as one of inference and optimization in a formally defined probability model – a joint row-column mixture model.

### Row-column mixture models

Consider a data matrix **X **where elements of the matrix are written as *x*_*i*,*j*_. In our model each row *i *of the matrix is associated with a row class random variable *r*_*i *_∈ {1,...,*n*_*r*_}, where *n*_*r *_is the number of possible row classes. Each column *j *of the matrix is associated with a column class random variable *c*_*j *_∈ {1,...,*n*_*c*_}, where *n*_*c *_is the number of column classes. The conditional distribution for element *x*_*i*,*j *_is then a function of the random variable associated with row *i *and column *j*. As such, the joint distribution of the data **X**, row classes *r*_*i *_and column classes *c*_*j *_can be written:

$$P(X,R,C)=\prod_{i}^{N_{r}}\prod_{j}^{N_{c}}p(x_{i,j}|r_{i},c_{j})P(r_{i})P(c_{j}).$$

Here we will use Gaussian models where $P(x_{i,j}|r_{i},c_{j})=N(x_{i,j};\Theta_{r_{i},c_{j}})$, where $\Theta_{r_{i},c_{j}}=\{\mu_{r_{i},c_{j}},\sigma_{r_{i},c_{j}}^{2}\}$ although other choices of distribution are possible. The unconditional distribution for each row class *r*_*i *_and column class *c*_*j *_is given by $P(r_{i})=\pi_{r_{i}}$ and $P(c_{j})=\pi_{c_{j}}$ respectively. Let all the row and column classes be written as $R=\{r_{1},...,r_{N_{r}}\}$, where *N*_*r *_is the number of rows in the matrix and let $C=\{c_{1},...,c_{N_{c}}\}$, where *N*_*c *_is the number of columns. It is insightful to contrast row-column mixtures with a traditional mixture of Gaussians for the rows of a matrix where the joint distribution for the data matrix and the row classes is given by: $P(X,R)=\prod_{i}\prod_{j}N(x_{i,j};\mu_{r_{i},j},\sigma^{2})\pi_{r_{i}}$, where $\mu_{r_{i},j}$ now represents elements of vectors ***μ***_*j*_. If, in the joint row-column model we assign each column to its own class then the models are equivalent.

### Variational optimization of row-column mixtures

In the following exposition we present a straightforward extension of the well known Expectation Maximization (EM) [35] approach to clustering for a joint row column mixture model. We then show how a simple adaptation of the more general algorithm leads to an approach that scales more easily to larger matrices. The algorithm we present here is a form of variational EM [36-38]. Traditional EM based clustering requires the posterior distribution over random variables for clusters. However, since we have associated random variables with *both *row and column clusters and these variables are conditionally dependent, traditional EM would require an intractable joint posterior distribution. We therefore use a tractable approximation to the posterior distribution *P*(*R*, *C*|**X**) consisting of

$$Q(R,C)=\prod_{i}Q(r_{i})\prod_{j}Q(c_{j}).$$

Starting with an initial guess for $\tilde{\Theta }$, it is possible to optimize a bound on the log probability of the observed data under our model by starting with initial guesses (e.g. uniform distributions) and iteratively updating *Q*(*r*_*i*_)s and *Q*(*c*_*j*_)s [37,38]. We can therefore optimize the parameters of our model by repeating the following two steps until updates converge to a final solution:

1. Variational E-steps for one or more rounds updating *Qs*, then

2. An M-step, updating $\tilde{\Theta }$.

To express our variational E-steps succinctly, define hidden row and column class membership or indicator variables as *H *= {*R*, *C*}. The variational updates for fully factorized *Q*s can be written

$$Q_{i}^{*}({\left\{H\right\}}_{k})=\exp(E_{Q_{l\neq k}}\langle \ln P(\tilde{X},H)\rangle ){\left(\sum_{{\left\{H\right\}}_{k}}\exp(E_{Q_{l\neq k}}\langle \ln P(\tilde{X},H)\rangle )\right)}^{-1}$$

where $E_{Q_{l\neq k}}\langle \cdot \rangle$ denotes the expectation under all *Q*_*l*≠*k *_and $\tilde{X}$ represents an observed data matrix **X**. Since one holds *Q*_*l*≠*k *_constant, these computations are performed locally in the graphical model corresponding to (1). Variables are updated in turn under random permutations of their ordering over iterations. The updates of parameters of the model are computed via a Maximization or M-step, setting $\frac{\partial }{\partial \theta }E_{Q}\langle \log P(\tilde{X},R,C)\rangle =0$, we obtain closed form updates for Θ. These updates are given by:

$$\mu_{r_{i},c_{j}}=\frac{\sum_{i}\sum_{j}Q(r_{i})Q(c_{j})x_{i,j}}{\sum_{i}\sum_{j}Q(r_{i})Q(c_{j})}$$

$$\sigma_{r_{i},c_{j}}^{2}=\frac{\sum_{i}\sum_{j}Q(r_{i})Q(c_{j}){\left(x_{i,j}-\mu_{r_{i},c_{j}}\right)}^{2}}{\sum_{i}\sum_{j}Q(r_{i})Q(c_{j})}$$

$$\begin{array}{cc} P(r_{i})=\frac{\sum_{i}Q(r_{i})}{N_{r}}, & P(c_{i})=\frac{\sum_{i}Q(c_{j})}{N_{c}} \end{array}$$

We refer to algorithm above as a variational optimization of a row column mixture (v-rc-mix). A closely related optimization method is to start with hard assignments for row and column classes and search for new Maximum a Posteriori (MAP) variable $\tilde{C}$ assignments by alternating updates for the assignments for all the rows ${\tilde{r}}_{i}$ and then all the columns ${\tilde{c}}_{j}$. One then interleaves updates of the parameter estimates. This is equivalent to constraining the *Q*(*r*_*i*_)s and *Q*(*c*_*j*_)s in (2) to take on a single value, the best possible hard assignment. Therefore the algorithm can be described with the same equations above and simply adding these constraints on the *Qs*. While this approach can fall into local minima more easily, for large matrices with row or column sizes exceeding hundreds of elements this hard assignment algorithm can have superior run time characteristics if implemented efficiently. We refer to the hard assignment version of the algorithm using (rc-mix).

### Sequential optimization of row-column mixtures

Our computational experiments suggest that the following algorithm has run-time and performance characteristics that are well suited to the size of matrices we explore here. For this reason we present the following *sequential optimization *algorithm. To optimize a row column mixture with this method we begin with a random hard assignment for row and column classes. We cycle through the rows and columns under a random permutation, removing the contribution of each row or column and compute the optimal class re-assignment *after *the parameters have also been updated. We therefore refer to this type of algorithm as a sequential optimization (s-rc-mix). We also note that for many applications in bioinformatics, the data matrix **X **may contain missing values. As such, the algorithm presented here formally deals with missing values. Specifically, when we count the number of elements *x*_*i*,*j *_within a *r *× *c *class, *N*_*r*,*c *_we do not include missing values. Under a hard assignment for row and column classes, the cost function we wish to minimize is given by

$$-E_{Q}\langle \log P(\tilde{X},R,C)\rangle =-\sum_{i}\sum_{j}\log P({\tilde{x}}_{i,j}|{\tilde{r}}_{i},{\tilde{c}}_{j})-\sum_{i}\log P({\tilde{r}}_{i})-\sum_{j}\log P({\tilde{c}}_{j}),$$

We then obtain an efficient update for our algorithm by observing that (7) can be re-written as the sum of terms $\sum_{k}^{n_{r}}\sum_{l}^{n_{c}}F_{k,l}$, where

$$\begin{array}{c} F_{k,l}=\sum_{i}\sum_{j}\delta_{{\tilde{r}}_{i},k}\delta_{{\tilde{c}}_{j},l}\left(\frac{{\left(x_{i,j}-\mu_{k,l}\right)}^{2}}{2\sigma_{k,l}^{2}}+\frac{1}{2}\log(2\pi \sigma_{k,l}^{2})\right)-\sum_{i}\delta_{{\tilde{r}}_{i},k}\log P({\tilde{r}}_{i})-\sum_{j}\sigma_{{\tilde{c}}_{j},l}\log P({\tilde{c}}_{j})\\ =\frac{1}{2\sigma_{k,l}^{2}}\left(\sum_{i}\sum_{j}\delta_{{\tilde{r}}_{i},k}\delta_{{\tilde{c}}_{j},l}x_{i,j}^{2}-N_{k,l}\mu_{k,l}^{2}\right)+\frac{N_{k,l}}{2}\log(2\pi \sigma_{k,l}^{2})-N_{r}\log P({\tilde{r}}_{i}=k)-N_{c}\log P({\tilde{c}}_{j}=l). \end{array}$$

Given this construction we can then optimize (7) by re-assigning rows and columns to new assignments under a random permutation with updates computed using the following equations. We remove a row or a column from its row or column class and compute the effect upon the parameters and each term in the cost function. The update of the parameters if a row *i *is removed from row class *k *can be written

$$\begin{array}{ccc} {\tilde{\mu }}_{k,l}=\frac{N_{k,l}\mu_{k,l}-\sum_{j}\delta_{{\tilde{c}}_{j},l}x_{i,j}}{{\tilde{N}}_{k,l}}, & {\tilde{\sigma }}_{k,l}^{2}=\frac{\tilde{N_{k,l}\bar{x_{k,l}^{2}}}-{\tilde{N}}_{k,l}{\tilde{\mu }}_{k,l}^{2}}{{\tilde{N}}_{k,l}}, & \tilde{P}(\tilde{r}=k)=\frac{{\tilde{N}}_{k}}{N_{r}} \end{array}$$

where ${\tilde{N}}_{k,l}=N_{k,l}-\sum_{j}\delta_{{\tilde{c}}_{j},l}$, ${\tilde{N}}_{k}=N_{k}-1$ and $\tilde{N_{k,l}\bar{x_{k,l}^{2}}}=N_{k,l}\bar{x_{k,l}^{2}}-\sum_{j}\delta_{{\tilde{c}}_{j},l}x_{i,j}^{2}$. The column update equations have the same form and the equations for adding a row or column also have a similar form.

### Comparing models and optimization algorithms

We wish to recover block structure within our matrix of expression levels for different anatomies and genes. Further, we wish to identify algorithms that will scale to high resolution, genomic scale data sets with hundreds of anatomical structures and thousands of genes. In the following experiments we investigate the difference between independently clustering rows and columns of matrices and different optimization methods for joint row-column mixture models. We compare k-means and Gaussian mixture models independently applied to the rows and columns of a matrix (i-mix) vs. optimizing row column mixture models with a hard assignment update algorithm (rc-mix), our variational update algorithm (v-rc-mix) and our sequential optimization method (s-rc-mix). We perform experiments recovering known block constant structure within synthetic data followed by experiments from a known DNA microarray data set, the mitogen activated protein kinase (MAPK) data of [39] for which the authors arranged a sub-matrix into block constant regions by hand. In both cases we compute recognition rates by assigning each row-column class to the dominant known class within the cluster. While other measures of cluster quality are possible, such recognition rates provide an intuitive measure of performance.

#### Synthetic data example

We generated 100 random matrices and present the mean and 95% confidence interval. To general matrices, with equal probability we choose a row-column mean of zero or a row-column mean drawn uniformly from the interval [-1, 1]. Means drawn from the mean zero class are given a variance drawn from the interval [0, .01] and for the means drawn from the random class we draw a variance uniformly from [0, .16]. Such matrices have similar statistics to our brain atlas data matrix and other DNA microarray experiments. Table 4 illustrates the results of tests recovering block constant structure from 50 × 50 element matrices with 5 row and 5 column classes.

**Table 4:** Comparing clustering algorithms (Left to Right) Recognition rates for: independently applied k-means, independent Gaussian mixture models (i-mix), alternating row and column hard assignment row-column mixtures (rc-mix), sequentially optimized row-column mixtures (s-rc-mix) and row-column mixtures optimized using our variational method (v-rc-mix). We recover the block constant structure of synthetic data consisting of 50 × 50 element matrices with 5 row and 5 column classes.

	k-means	i-mix	rc-mix	s-rc-mix	v-rc-mix
recognition rate	.71 ± .04	.72 ± .04	.77 ± .04	.83 ± .04	.84 ± .05
time (sec.)	.15 ± .02	.22 ± .01	1.0 ± .06	2.3 ± .2	110 ± 10

#### DNA microarray example

In these experiments we compare the algorithms described in the previous sections for the task of automatically determining the class assignments that were used to create figure 14b within [39]. In this figure, a subset of 36 experiments for 67 genes were grouped into 7 row and 8 column cluster blocks "by hand" *after *an initial hierarchical clustering of a larger 46 × 400 microarray data matrix. Importantly, these hand assigned row column clusters indeed exhibit approximately block constant structure. For the following experiments we randomize this matrix, apply the algorithms described in the previous section and use the hand labeled class assignments as the *ground truth*. Table 5 illustrates the recognition rates and timing results for this data set using the different algorithms when we treat the dominant known class in a cluster as the true label.

**Table 5:** Results for recovering the block constant structure in the MAPK microarray data [39]. (Left to Right) Recognition rates for: independently applied k-means, independent Gaussian mixture models (i-mix), alternating row and column hard assignment row column mixtures (rc-mix), sequentially optimized rc-mixtures (s-rc-mix) and variational optimization for rc-mixtures (v-rc-mix). We recover known, approximately block constant structure in a 36 × 67 element matrix with 7 row and 8 column classes.

	k-means	i-mix	rc-mix	s-rc-mix	v-rc-mix
recognition rate	.50 ± .01	.48 ± .01	.56 ± .01	.56 ± .01	.55 ± .01
time (sec.)	.38 ± .01	.46 ± .01	3.8 ± .1	4.6 ± .4	195 ± 3

#### Modeling and computational considerations

First, when performing joint row-column cluster analysis one must of course have reason to believe that block constant structure exists in the underlying data. However, figure 11 indicates that these model assumptions are reasonable for this subset of the Allen Atlas data. Second, our performance and runtime analysis suggests that for matrices of approximate size 50 × 50 our variational optimization procedure is an attractive approach. For matrices of approximate size 200 × 200 the computation time for our variational method is such that our sequential optimization algorithm becomes more attractive. Finally, for matrices with several hundred anatomical structures and several thousand genes, given a fixed amount of time for computation, the alternating row column greedy optimization algorithm has desirable characteristics. Finally, for future work in this area we see annealing approaches and automated model selection methods as having great potential for these types of models.

## Competing interests

The authors declare that they have no competing interests.

## Authors' contributions

MJ performed the image processing and functional enrichment analysis. CP conceived of and implemented the block clustering analysis. CP, ELM and DK directed the research and contributed to the writing of the manuscript. RTZ provided information on mouse neurobiology.
